# Aufnahmediagnose Prellung: Ätiologie, Epidemiologie und Kostenfaktoren

**DOI:** 10.1007/s00391-020-01828-w

**Published:** 2020-12-18

**Authors:** Mathias Woschek, Cora R. Schindler, Jasmina Sterz, Philipp Störmann, Laurent Willems, Ingo Marzi, René D. Verboket

**Affiliations:** 1 Klinik für Unfall‑, Hand- und Wiederherstellungschirurgie, Goethe Universität Frankfurt, Theodor-Stern-Kai 7, 60590 Frankfurt am Main, Deutschland; 2 Epilepsiezentrum Frankfurt Rhein-Main und Klinik für Neurologie, Goethe Universität Frankfurt, Frankfurt am Main, Deutschland

**Keywords:** Notaufnahme, Bagatelltrauma, Evidenzbasierte Medizin, Vergütung, Gesundheitsökonomie, Emergency room, Minor injury, Evidence based medicine, Remuneration, Health economics

## Abstract

**Hintergrund:**

Die stationäre Aufnahme von Patienten mit Prellungen wird in Kliniken der Akutversorgung regelhaft praktiziert. Dabei stehen die pathophysiologischen Unfallfolgen oft im Hintergrund. Ziel dieser retrospektiven monozentrischen Untersuchung war die Untersuchung der Ätiologie sowie der kostenverursachenden Faktoren und Refinanzierung bei Aufnahmen durch Prellungen.

**Methodik:**

Es erfolgte die Abfrage der Patienten entsprechend den Entlassdiagnosen aus dem krankenhausinternen Informationssystem (KIS). Eingeschlossen wurden 117 Patienten in einem Zeitraum von 2 Jahren. Es erfolgten hier die Klassifizierung nach Unfallmechanismus sowie die Einteilung in Altersgruppen. Des Weiteren erfolgte die Kostenkalkulation anhand von abteilungs- und klinikspezifischen Tagessätzen.

**Ergebnisse:**

Bezüglich der Ätiologie war der häusliche Sturz die häufigste Ursache (48,7 %), gefolgt von dem Hochrasanztrauma (22,8 %). Innerhalb der Gruppe des häuslichen Sturzes lag das Durchschnittsalter im Mittel bei 77,8 Jahre. Diese Gruppe zeigte die längste Verweildauer (VWD) mit 5,2 Tagen. Im Rahmen der kalkulierten Kosten zeigte die Gruppe nach häuslichem Sturz die höchsten Kosten mit 2596,24 € bei einem mittleren DRG-Erlös von 1464,51 €.

**Diskussion:**

Die Auswertung der klinikinternen Daten bestätigte die subjektive Wahrnehmung, dass ein Großteil der nach Prellung aufgenommenen Patienten aus der Altersgruppe >65 Jahre stammt. Die Aufnahme erfolgt hier vor dem Hintergrund der in dieser Altersgruppe zunehmenden Komorbiditäten sowie zur Abwendung von Folgeerkrankungen und Folgen der Immobilisierung. Ebenfalls konnte gezeigt werden, dass die Versorgungskosten gesundheitsökomisch relevant sind und die Behandlung in diesen Fällen nicht kostendeckend ist.

## Abstract (en)

**Background:**

The admission of patients with minor injuries, such as contusions is a regular practice in acute care hospitals. The pathophysiological changes resulting from the accident are seldom the primary reason for hospitalization. The aim of this retrospective monocentric study was therefore to examine the etiology as well as the cost-causing factors and refinancing on admission.

**Methods:**

Patients were identified due to a retrospective query in the hospital information system (HIS) according to the ICD-10 German modification codes at discharge. A total of 117 patients were enrolled over a period of 2 years. The classification was carried out according to the accident mechanism and the division into age groups. In addition, the cost calculation was based on department and clinic-specific daily rates.

**Results:**

In terms of etiology low impact falls in the domestic environment were the most common cause (48.7%), followed by high-energy trauma (22.8%). Within the group with domestic falls, the mean age was 77.8 years. This group also showed the longest length of stay (LOS) with 5.2 days. As part of the calculated costs, the group of domestic falls showed the highest costs of 2596.24 € with an average DRG cost revenue of 1464.51 €.

**Discussion:**

The evaluation of the clinic internal data confirmed the subjective perception that the majority of patients admitted with the diagnosis of contusions came from the age group >65 years. Admission is primarily based on the increasing comorbidities and to avert secondary diseases and the consequences of immobilization. It could also be shown that the resulting costs are relevant to health economics and that the treatment does not appear to cover the costs.

Prellungen bilden einen relevanten Anteil der in einer chirurgischen Notaufnahme gestellten Diagnosen. Während bei jungen gesunden Patienten oft eine ambulante Behandlung ausreichend ist, führen Prellungen im erhöhten Lebensalter trotz fehlenden pathophysiologischen Äquivalenten zu einer deutlichen Einschränkung der Mobilität und Selbstständigkeit. Dies resultiert häufig in einer stationären Aufnahme der Patienten. Ziel dieser Arbeit ist es, die Ätiologie sowie die beeinflussenden Faktoren auf die Gesamtverweildauer bei stationärer Aufnahme zu untersuchen. Des Weiteren erfolgt eine Kostenanalyse mit der Frage nach ausreichender Refinanzierung der stationären Behandlung in diesen Fällen.

## Einleitung

Die Vorstellung von Patienten mit Prellungen macht einen relevanten Anteil der Arbeit in zentralen Notaufnahmen chirurgischer Zentren aus. Prellungen sind häufig durch eine stumpfe Gewalteinwirkung gekennzeichnet. In einer abgestuften Weise, als direkte Krafteinwirkung mit verschiedenen Schweregraden, wird die Prellung von der Kontusion und der Quetschung unterschieden. Bei der Prellung liegen histopathologisch keine schwerwiegenden Gewebeschädigungen in Form von Nekrosen vor; klinisch dominiert der Schmerz [1]. In der Praxis haben sich die Diagnosen Prellung für banale Schäden ohne längerfristige Relevanz und Kontusion für stärkere Prellungen mit schwerwiegenden Befunden durchgesetzt; die Quetschung stellt die maximale Ausprägung dieser Verletzungen dar [1].

Während ein Großteil dieser Patienten nach erfolgtem Frakturausschluss ambulant behandelt wird, kommt es regelhaft auch zur stationären Aufnahme. Gründe hierfür sind das Alter der Patienten sowie assoziierte Komorbiditäten wie Demenz und chronische Erkrankungen [2, 3]. Die Aufnahme von Patienten mit Bagatellverletzungen führt jedoch zur Belegung von für die Akutbehandlung notwendigen Betten und bindet einen Großteil der innerklinischen Reserven, ohne dass dies ausreichend refinanziert wird [4].

Im Hinblick auf die Ursache der Verletzungen zeigt sich der Sturz aus geringer Höhe als führend und macht damit etwa 45 % der gesamten Aufnahmen einer unfallchirurgischen Notaufnahme aus [5]. Mit zunehmendem Lebensalter erhöht sich auch die Inzidenz für diese Entität [6–8]. Ein Drittel der Patienten mit einem Lebensalter über 65 Jahre stürzt statistisch einmal pro Jahr; dieser Anteil steigt in der Gruppe über 80 Jahre auf mehr als 50 % an [9].

Basierend auf den Daten des deutschen Bundesamtes für Statistik und Vorhersagemodellen wird der Anteil der älteren Bevölkerung in den kommenden Jahren zunehmen; dieser demografische Wandel spiegelt sich auch in den Einrichtungen des öffentlichen Gesundheitswesens wider [10]. Modelle gehen hier bis zum Jahre 2030 von einer Zunahme auf bis zu 25 % der Gesamtbevölkerung aus [11]. Hieraus ergeben sich sowohl eine zunehmende soziale als auch ökonomische Belastung des Gesundheitssystems [12, 13].

Ziel dieser Arbeit war es, objektive Daten für die stationär behandelten Patienten mit Prellungen der oberen und unteren Extremität sowie des Körperstammes hinsichtlich der Ätiologie des Traumas sowie der Länge des stationären Aufenthaltes („length of stay“, LOS) und der beeinflussenden Faktoren zu untersuchen. Zudem werden kostenverursachende Faktoren untersucht und mögliche Vergütungsprobleme des aktuellen Abrechnungssystems nach G‑DRG analysiert.

## Studiendesign und Methoden

### Studiendesign und Datenakquise

Diese Untersuchung stellt die Daten einer retrospektiven Studie zu Patienten dar, die mit der Diagnose Prellung zwischen dem 01.01.2018 und dem 31.12.2019 in der Abteilung für Unfall‑, Hand- und Wiederherstellungschirurgie am Universitätsklinikum Frankfurt am Main stationär aufgenommen wurden.

**Figure 1:**
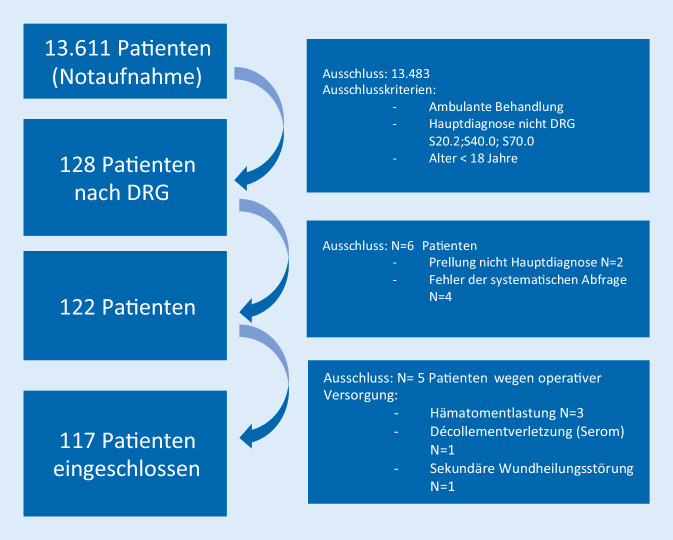

Eingeschlossen wurden Patienten die infolge einer Behandlung in der Notaufnahme zur stationären Therapie aufgenommen und mit den DRG-Codierungen S20.2 (Prellung des Thorax), S40.0 (Prellung von Schulter und Oberarm) und S70.0 (Prellung der Hüfte) als Hauptdiagnosen entlassen worden sind. Die Behandlungsdaten sowie die Haupt- und Nebendiagnosen wurden mittels systematischer Abfrage im Krankenhausinformationssystem (KIS) erhoben. Eingeschlossen wurden Patienten im Alter >17 Jahre (Abb. 1). Mittels Durchsicht der Aufnahmeprotokolle wurden alle Patienten identifiziert und in Gruppen nach Unfallmechanismus eingeteilt (Hochrasanztrauma nach DGU-S3-Leitlinie, häuslicher Sturz (Sturzhöhe unter 1,5 m), Z. n. Gewalttat, Sturz aus >3 m Höhe, Fahrradsturz) [14]. In die Gruppe Sonstige wurden Patienten, die zu keiner der vorgenannten Gruppen passen, einsortiert; durch die Heterogenität der Gruppe ist diese für die Gesamtberechnung relevant, jedoch nicht zum Gruppenvergleich geeignet. Die vorliegende Studie ist durch die lokale Ethikkommission genehmigt (Frankfurt am Main, Genehmigung 19-491) und folgt den STROBE-Richtlinien für Beobachtungsstudien (Strengthening The Reporting of Observational Studies in Epidemiology) sowie den RECORD-Richtlinien für Observationsstudien (Reporting of studies Conducted using Observational Routinely-collected Data) [15, 16].

### Kostenkalkulation

Entsprechend den aktuellen Empfehlungen zur Kalkulation von stationären Behandlungskosten kann neben den DRG-Erlösen auch eine Berechnung anhand von abteilungs- und klinikspezifischen Tagessätzen erfolgen [17, 18]. Pauschalen von 442,21 € für Normalstationen [17], welche Pflege, Medikation und Diagnostik beinhalten, wurden berechnet. Analog zu anderen gesundheitsökonomischen Analysen wurden die Pauschalen um die Inflation im medizinischen Sektor korrigiert [19–21].

### Datenverarbeitung und Statistik

Die Aufarbeitung und statistische Auswertung der Daten erfolgte unter Verwendung von IBM SPSS Statistics 25 (SPSS Inc., Chicago, IL, USA). Die statistische Aufarbeitung der DRG-Erlöse erfolgte aufgrund einer rechtsschiefen Verteilung der anfallenden Kosten unter Verwendung der Bootstrap-Methode [22]; es erfolgt die Angabe von Mittelwert, Standardabweichung, Minimum, Maximum, Median sowie des 95 %-Konfidenzintervalls. Die univariate Analyse von mit höheren Kosten assoziierten Faktoren erfolgte unter einheitlicher Verwendung des Eta-Koeffizienten (η) bei Verwendung je eines metrisch- (Kosten) und eines nominalskalierten Parameters. Aus der Berechnung resultiert ein Koeffizient zwischen 0 (keine Korrelation) und 1 (sehr starke Korrelation). Hierbei zeigen ein η < 0,01 keine Korrelation an, ein η zwischen 0,01 und 0,04 eine geringe, ein η zwischen 0,04 und 0,16 eine mittelstarke und ein η von >0,16 eine starke Korrelation [23]. Zudem erfolgt die Angabe von Somers’ d zur Analyse einer signifikanten Korrelation [23–25]. Die univariate Analyse hinsichtlich potenzieller Vergütungsprobleme erfolgte in gleicher Weise, basierend auf der Differenz zwischen kalkulierten Kosten und tatsächlichem Erlös. Alle Ergebnisse wurden nach der Benjamini-Hochberg Korrektur für multiples Testen korrigiert [26].

## Ergebnisse

**Figure 2:**
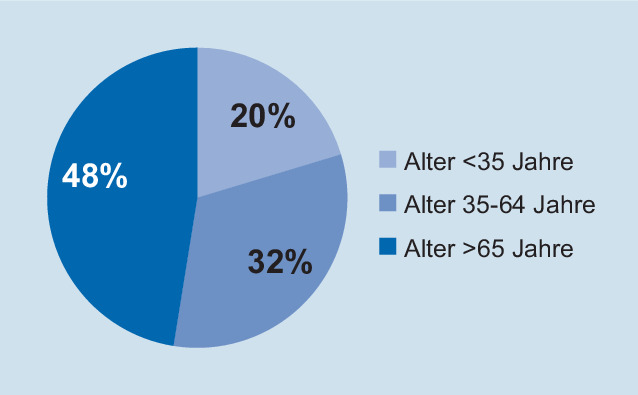

Über das KIS konnten 117 Patienten, welche zwischen dem 01. Januar 2018 und dem 31. Dezember 2019 mit einer Prellung über unsere zentrale Notaufnahme stationär aufgenommen wurden, identifiziert werden. Es zeigte sich eine vornehmlich männliche (54,7 %, 64/117) Kohorte mit einem Altersmittelwert von 61,1 Jahren (SD ± 23,35 Jahre, Min: 18 Jahre, Max: 97 Jahre). Zwanzig Prozent der untersuchten Patienten waren unter 35 Jahre alt, 32 % im Alter von 35 bis 64 Jahren und 48 % waren über 65 Jahre alt (Abb. 2). Bei Patienten unter 65 Jahren zeigten 59,7 %, in der Patientengruppe größer 65 Jahre 96,3 % eine oder mehr Komorbiditäten.

### Unfallmechanismen und Altersverteilung

**Figure 3:**
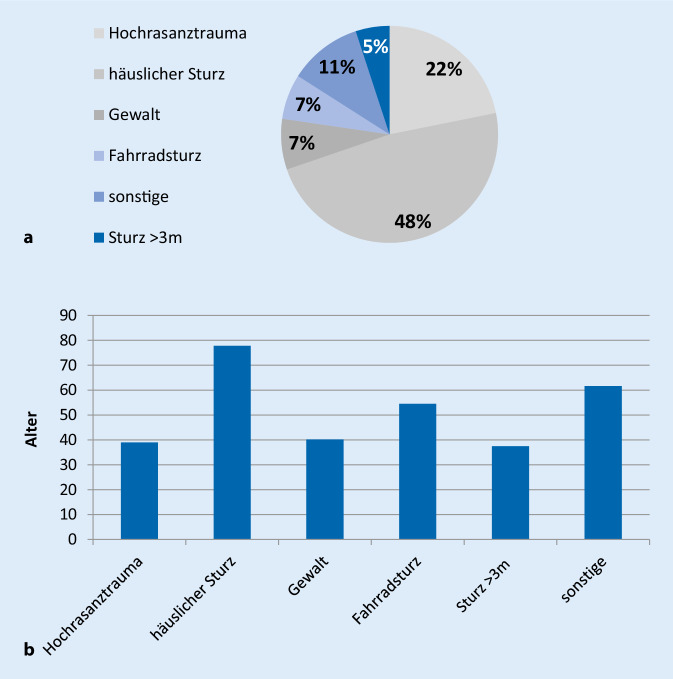

Es zeigte sich eine gehäufte Vorstellung von Patienten mit Prellungen nach häuslichem Sturz (48,7 %, 57/117), gefolgt von Patienten nach Hochrasanztrauma (22,2 %, 26/117). Kleinere Anteile entfielen auf Patienten nach Gewalttaten (7,7 %, 9/117), Fahrradstürzen (6,8 %, 8/117) oder Stürzen aus >3 m Höhe (5,1 %, 6/117) (Abb. 3a). Betrachtet man die Altersverteilung in den Gruppen, lässt sich der höchste Altersmittelwert (MW) in der Gruppe nach häuslichem Sturz feststellen (MW: 77,8; SD ± 14,8). Im Mittelfeld der Altersgruppierung finden sich Patienten nach Fahrradsturz (MW: 54,5; SD ± 17,1). Eher jüngere Patienten fanden sich in den Gruppen Sturz aus >3 m Höhe (MW: 37,5; SD ± 13,6), Hochrasanztrauma (MW: 39,0; SD ± 13,8) und Z. n. häuslicher Gewalt (MW: 40,2; SD ± 17,0) (Abb. 3b).

### Verweildauer der Patienten

**Figure 4:**
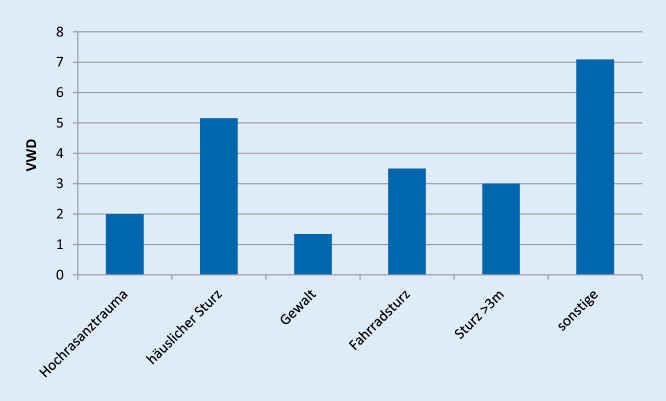

Betrachtet man die Verweildauer (VWD) nach Unfallmechanismus zeigt sich die längste VWD in Tagen in der Gruppe nach häuslichem Sturz (MW: 5,2; SD ± 3,4), gefolgt von der Gruppe nach Fahrradsturz (MW: 3,5; SD ± 3,1). Eine kürzere VWD zeigte sich in der Gruppe nach Sturz aus >3 m Höhe (MW: 3,0; SD ± 2,0) und nach Hochrasanztrauma (MW: 2,0; SD ± 2,0). Die kürzeste VWD zeigte sich in der Gruppe mit Zustand nach Gewalttat (MW: 1,3; SD ± 0,5) (Abb. 4).

**Figure 5:**
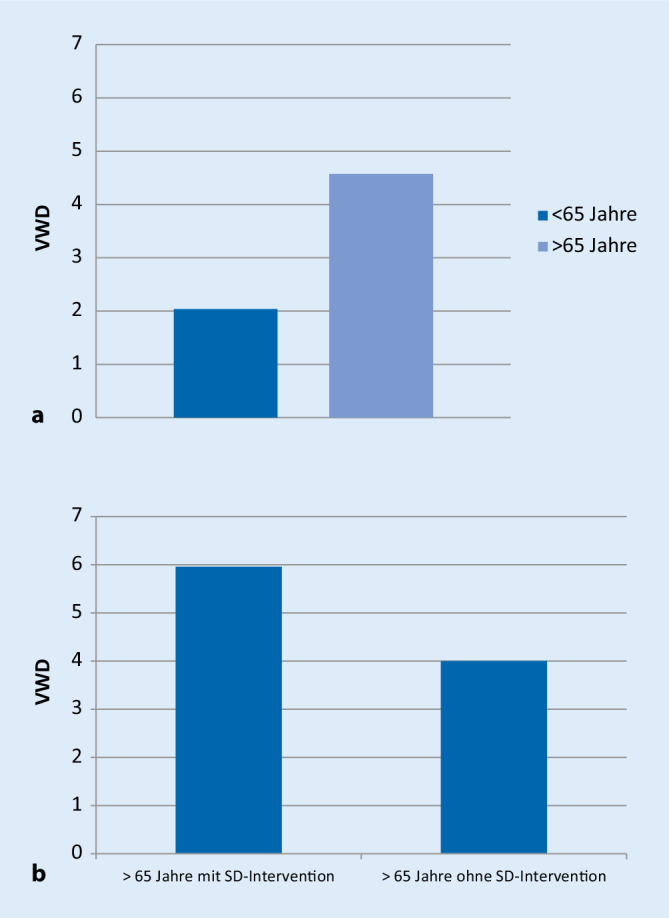

Unterscheidet man die Gesamtverweildauern nach Patienten unter 65 Jahre und über 65 Jahre zeigt sich in der Gruppe der über 65 jährigen eine deutlich höhere VWD (MW 4,57; SD ± 4,01) als in der Gruppe der unter 65 jährigen (MW 2,04; SD ± 3,17) (*p* < 0,05) (Abb. 5a).

Bei den Patienten über 65 Jahre zeigt sich bei Sozialdienstintervention eine verlängerte VWD mit 5,96 (SD ± 3,34) vs. 4 Tagen (SD ± 5,03) (*p* = 0,085) (Abb. 5b). Eine Sozialdienstintervention war in 25 Fällen notwendig; das Zeitintervall zwischen Aufnahme und Initiierung der Sozialdienstintervention lag bei 1,92 Tagen (SD ± 0,74).

### Tatsächlicher Erlös, kalkulierte Kosten und kostenverursachende Faktoren

Der tatsächliche mittlere DRG-Erlös der analysierten Fälle betrug 1449,20 € (SD ± 724,92 €) (Tab. 1).

**Table 1:** 

Kollektiv	Einzelposten	Kostenberechnung					
		MW (€)	± SD	Med (€)	Min (€)	Max (€)	95 %-KI (€)
Alle Patienten	Kosten, stationärer Aufenthalt	1825,53	1638,91	1326,63	442,21	9286,41	1528,57–2122,50
	Gesamtkosten	2198,45	1772,48	1640,50	684,76	10276,02	1877,28–2519,62
	DRG-Erlös	1449,20	724,92	1464,51	816,40	4306,55	1317,85–1580,56
Patienten nach Hochrasanztrauma	Kosten, stationärer Aufenthalt	884,42	892,88	442,21	442,21	4422,1	541,21–1227,63
	Gesamtkosten	1177,45	978,36	693,68	684,76	5090,8	801,39–1553,51
	DRG-Erlös	1139,75	562,70	859,18	859,18	3028,43	923,46–1356,04
Patienten nach häuslichem Sturz	Kosten, stationärer Aufenthalt	2288,63	1493,58	2211,05	442,21	7075,36	1900,89–2676,37
	Gesamtkosten	2686,84	1614,91	2596,24	684,76	7852,81	2267,60–3106,07
	DRG-Erlös	1604,90	578,68	1738,51	816,41	2941,82	1454,67–1755,13
Patienten nach Gewalttat	Kosten, stationärer Aufenthalt	589,61	208,46	442,21	442,21	884,42	453,42–725,80
	Gesamtkosten	886,87	215,95	789,97	684,76	1204,62	745,78–1027,95
	DRG-Erlös	823,03	13,24	816,41	816,41	856,14	814,38–831,68
Patienten nach Sturz aus >3 m Höhe	Kosten, stationärer Aufenthalt	1326,63	884,42	884,42	442,21	3095,47	618,96–2034,30
	Gesamtkosten	1686,21	980,97	1208,39	702,59	3657,19	901,28–2471,14
	DRG-Erlös	1128,11	440,87	816,41	816,41	1764,51	775,34–1480,87
Patienten nach Fahrradsturz	Kosten, stationärer Aufenthalt	1547,74	1380,80	884,42	442,21	4864,31	590,91–2504,56
	Gesamtkosten	1927,60	1484,78	1192,54	789,97	5512,98	898,73–2956,48
	DRG-Erlös	1408,04	826,58	816,41	816,41	3028,43	835,26–1980,83
Patienten mit sonstigem Grund für Prellungen	Kosten, stationärer Aufenthalt	3135,67	2629,40	2211,05	442,21	9286,41	1581,82–4689,52
	Gesamtkosten	3630,45	2849,40	2638,23	684,76	10276,02	1946,60–5314,31
	DRG-Erlös	2091,25	1166,30	1640,50	816,40	4306,55	1402,02–2780,48

*MW* Mittelwert, *SD* Standardabweichung, *Med* Median, *Min* Minimum, *Max* Maximum, *KI* Konfidenzintervall

In der Kalkulation der Behandlungskosten zeigte sich im Mittel über alle Patienten ein Betrag von 2198,45 € (SD ± 1772,48 €), wobei der größte Anteil der Kosten für den stationären Aufenthalt anfiel (1825,53 € ± 1638,91 €). Höhere kalkulierte Kosten konnten in der Gruppe der Patienten nach häuslichem Sturz (2686,84 € ± 1493,58 €) gegenüber dem Gesamtkollektiv berechnet werden, hier begründen sich die höheren Kosten v. a. im längeren Verbleib auf der Normalstation (Tab. 1). Anhand dieser Berechnungen konnte die Analyse kostenverursachender Faktoren erfolgen. Hier zeigte sich eine stark signifikante Korrelation nach multiplem Testen bei Patienten mit Alter über 65 Jahre (*p* < 0,001), Patienten nach Hochrasanztrauma (*p* < 0,001), Patienten nach häuslichem Sturz (*p* < 0,001) und Patienten nach Gewalttat. (*p* = 0,012) (Tab. 2). Ebenso zeigte sich eine signifikante, starke Korrelation von Diskrepanzen zwischen kalkulierten Kosten und tatsächlichem Erlös in Bezug auf Patienten mit Alter über 65 Jahre (*p* < 0,001), Patienten nach Hochrasanztrauma (*p* < 0,001), Patienten nach häuslichem Sturz (*p* < 0,001) und Patienten nach Gewalttat (*p* = 0,03) (Tab. 2).

**Table 2:** 

Faktor	Kohorte		Kostenverursachende Faktoren			Potenzielle Faktoren für Vergütungsprobleme		
		% (*n*)	η^a^	D^a^	*p*-Wert	η^a^	D^a^	*p*-Wert
Demografie								
Geschlecht	W	45,3 (53)	0,037	−0,029	0,787	0,07	−0,101	0,5733
	M	54,7 (64)	–	–	–	–	–	–
Alter	<65	53,8 (63)	0,352	0,461	**<0,001**	0,324	0,47	**<0,001**
	>65	46,2 (54)	–	–	–	–	–	–
Trauma								
Patienten nach Hochrasanztrauma	Ja	22,2 (26)	0,308	−0,594	**<0,001**	0,247	−0,45	**<0,001**
	Nein	77,8 (91)	–	–	–	–	–	–
Patienten nach häuslichem Sturz	Ja	48,7 (57)	0,269	0,423	**<0,001**	0,239	0,444	**<0,001**
	Nein	51,3 (60)	–	–	–	–	–	–
Patienten nach Gewalttat	Ja	7,7 (9)	0,214	−0,504	**0,012**	0,186	−0,636	**0,03**
	Nein	92,3 (108)	–	–	–	–	–	–
Patienten nach Sturz aus >3 m Höhe	Ja	5,1 (6)	0,067	−0,015	0,932	0,059	0,030	0,881
	Nein	94,9 (111)	–	–	–	–	–	–
Patienten nach Fahrradsturz	Ja	6,8 (8)	0,041	−0,018	0,917	0,06	−0,126	0,697
	Nein	93,2 (109)	–	–	–	–	–	–
Patienten mit sonstigem Grund für Prellungen	Ja	9,4 (11)	0,260	0,408	**0,048**	0,21	0,218	0,573
	Nein	90,6 (106)	–	–	–	–	–	–

Signifikante Werte (p < 0,05) wurden zur besseren Übersicht hervorgehoben

## Diskussion

Das von uns untersuchte Patientenkollektiv zeigte hinsichtlich der Verletzungsursache und der Altersverteilung eine Kohärenz zu anderen Untersuchungen. So zeigt z. B. die Untersuchung von Maegele et al. (2014) eine trimodale Verteilung im Hinblick auf die altersabhängige Inzidenz des Schädel-Hirn-Traumas [27]. Während in der Gruppe der jungen Patienten (<65 Jahre) die Ursachen führend im Bereich der Hochrasanztraumata sowie der Berufsunfälle liegt, zeigen die Patienten mit höherem Lebensalter sich im Zusammenhang mit Stürzen [9, 27].

In Bezug auf die VWD zeigt sich in unseren Daten eine signifikante Zunahme in der Gruppe der Patienten mit einem Lebensalter >65 Jahren. Diese Daten zeigen sich im Einklang mit internationaler Literatur; so zeigten Bergeron et al. [28] in einer registerbasierten Auswertung, dass ein Patientenalter >65 Jahre nach einem Sturzgeschehen mit einem verlängerten Krankenhausaufenthalt, einem erhöhten Risiko für die Unterbringung in Pflegeeinrichtungen sowie einer erhöhten Mortalität einhergeht [28]. Für Patienten mit Prellungen konnte dies ebenso wie eine erhöhte Mortalität oder Funktionsverluste noch nicht gezeigt werden.

Betrachtet man den Sturz als auslösenden Faktor, so zeigen diverse Studien, dass eine positive Sturzanamnese innerhalb eines Jahres ein positiver prädiktiver Faktor für eine zunehmende funktionelle Einschränkung und für eine wiederholte Hospitalisierung ist [29].

Die oben aufgeführten Diagnosen sowie die geringe Verletzungsschwere fungieren oft als Trigger einer ökonomischen Prüfung der Krankenhausbehandlung. Gegenstände dieser Prüfung sind hier der Verdacht der primären bzw. sekundären Fehlbelegung sowie die Prüfung einer sachgerechten Endgeldzuordnung [30]. Die primäre Fehlbelegung beschreibt hier eine unter stationären Bedingungen erbrachte Leistung, welche auch ambulant durchgeführt werden kann. Die sekundäre Fehlbelegung gesteht zwar eine stationäre Krankenhausbehandlung ein, stellt jedoch die Dauer der Behandlung infrage [30]. Die durchgeführte Analyse in Zusammenschau mit den Daten des deutschen Statistischen Bundesamtes zeigen, dass die Kliniken immer öfter in ein ethisch-ökonomisches Dilemma geführt werden. Die größer werdende Anzahl von alleinlebenden Menschen in unserer Gesellschaft mit einem Lebensalter >65 Jahren wird diese Belastung zunehmend verstärken [31].

Entsprechend den Indikationen für einen stationären Aufenthalt zeigen sich die fehlende Selbstversorgung und die Abwendung weiteren Schadens als zentrale Bestandteile. Im Rahmen von Prüfungen wird die VWD oft als Qualitätsindikator der stationären Behandlung gesehen, dass grade diese in Bezug auf ältere Patienten nach Stürzen kein Zeichen für Qualität ist, zeigen Auswertungen von Kennedy et al. [5].

Wichtiger Bestandteil der Therapie von älteren Patienten ist führend die Mobilisation unter physiotherapeutischer Anleitung; hierfür ist eine deutlich höhere Frequenz und Intensität erforderlich, als es der ambulante Sektor abzudecken vermag [32]. Gute Ergebnisse leistet hier die geriatrische Komplexbehandlung [33, 34]. Ein weiterer Punkt der Versorgung entfällt auch auf den Sozialdienst und die Organisation von häuslicher Unterstützung, Hilfsmitteln oder einer Anschlussbehandlung. So zeigen auch die von uns analysierten Daten eine signifikante Verlängerung der stationären VWD in der Gruppe der Patienten, die eine Mitbetreuung durch den Sozialdienst benötigten. Die Struktur der Krankenhäuser in Deutschland zeigt sich zunehmend im Rahmen von Netzwerken organisiert. Hierbei stellen Krankenhäuser der Maximalversorgung einen Knotenpunkt dar. Um diese Rolle erfüllen zu können, muss ein entsprechender Patientenfluss realisiert werden. Die von uns erhobenen Daten zeigen Faktoren einer verlängerten und zudem nicht kostendeckend durchgeführten Behandlung von Patienten mit Prellungen. Dies führt zu einer Belegung von notwendigen Bettenkapazitäten in der Akutbehandlung. Zwar findet ein Patientenstrom von Kliniken geringerer Versorgungsmöglichkeiten hin zum Maximalversorger statt, jedoch keiner von Patienten mit leichten Verletzungen in die Peripherie dieses Netzwerkes. Ein Grund dafür ist die oben aufgeführte mangelhafte Kostendeckung der Behandlung. Während die Aufnahme von schwer verletzten oder in der operativen Versorgung aufwendigen Patienten für den Maximalversorger eine Anhebung des CMI (Case-Mix-Index) und dadurch auch eine ökonomische Verbesserung darstellt, wäre ein umgekehrter Vorgang, basierend auf unseren Daten, mit einer finanziellen und pflegerischen Mehrbelastung der Krankenhäuser der Regel- und Grundversorgung vergesellschaftet. Unter dem vorherrschenden ökonomischen Druck kam es in der Krankenhausstruktur in Deutschland zu einer Reduktion der stationären Betten- und Behandlungskapazitäten. Waren 2006 noch 510.767 Betten in 2104 Kliniken vorhanden, so reduzierte sich diese Zahl bis zum Jahr 2016 auf 498.718 Betten in 1951 Kliniken [35]. Bei steigenden Fallzahlen kann die Patientenversorgung nur durch eine Verringerung der VWD und eine erhöhte Bettenauslastung gewährleistet werden. Um die hohen Fallzahlen bedienen zu können, ist eine zeitnahe Entlassung oder Anschlussheilbehandlung der Patienten notwendig. Diese erfolgt führend über rehabilitative Einrichtungen. Betrachtet man die Bettenentwicklung in diesen Kliniken, so zeigt sich hier jedoch eine äquivalente Situation. Im Jahr 2006 standen deutschlandweit 1255 Kliniken mit 172.717 Betten zur Verfügung; im Jahr 2016 reduzierte sich sowohl die Anzahl der Kliniken als auch die Anzahl der Betten auf 1149 Kliniken mit 165.223 Betten; während sich also die durchschnittliche VWD kaum änderte, stieg die Fallzahl um 8 % an. Dies führt zu einer deutlichen erhöhten Auslastung der verfügbaren Betten mit einer Zunahme um 11,3 % auf 83,0 % und den damit verbundenen Wartezeiten auf einen Weiterbehandlungsplatz [35].

Obwohl diese Studie unter Beachtung der STROBE- und RECORD-Richtlinien für Beobachtungsstudien bzw. Observationsstudien durchgeführt wurde [15, 16], erscheinen einzelne Punkte als Limitationen. Durch die Auswahl eines monozentrischen, retrospektiven Studiendesigns für die Jahre 2018 und 2019 ist die Anzahl der in die Studie aufgenommenen Patienten relativ begrenzt. Da jedoch nur eine orientierende Schlussfolgerung getroffen wird, und aufgrund der einheitlichen Vergütung stationärer Leistungen in Deutschland nach G‑DRG sowie der standardisierten, SOP- und leitlinienorientierten Behandlung erscheint ein Vergleich der Ergebnisse mit anderen Kliniken auf nationaler Ebene, wie auch schon in anderen Studien gezeigt, vertretbar [4, 36–40]. Eine Analyse von Vorerkrankungen als kostenverursachender Faktor in diesem Setting konnte auf aufgrund der Patientenzahl und der vielen verschiedenen möglichen Vorerkrankungen nicht getroffen werden; die Endgruppengrößen waren zu klein. Da diese jedoch gerade in der Gruppe >65 Jahre einen großen Teil ausmachen und sich insbesondere auch auf die Liegedauer auswirken können, können Vorerkrankungen theoretisch einen weiteren Faktor für Vergütungsprobleme darstellen [28, 29, 41].

## Schlussfolgerung

Die Versorgungskosten bei Patienten, die mit Prellungen aufgenommen werden, sind hoch und gesundheitsökonomisch relevant; oft handelt es sich um ältere Patienten, bei denen, wenn sie nicht betreut würden, eine Immobilisierung und Folgeerkrankungen drohen. Dem kann nur durch frühes geriatrisches Assessment und weitere geriatrische Versorgungsstrukturen entgegengewirkt werden. Es stellt sich insbesondere der längere stationäre Aufenthalt, oft kombiniert mit einer Sozialdienstinteraktion, als kostenverursachender Faktor dar. Generell scheint die auf Fallpauschalen basierende Vergütung der Kosten von stationären älteren Patienten mit Prellungen nicht kostendeckend.
